# Both attention and prediction are necessary for adaptive neuronal tuning in sensory processing

**DOI:** 10.3389/fnhum.2014.00152

**Published:** 2014-03-26

**Authors:** Yi-Fang Hsu, Jarmo A. Hämäläinen, Florian Waszak

**Affiliations:** ^1^Université Paris Descartes, Sorbonne Paris CitéParis, France; ^2^CNRS, Laboratoire Psychologie de la Perception, UMR 8242Paris, France; ^3^Department of Psychology, University of JyväskyläJyväskylä, Finland

**Keywords:** attention, prediction, sensory processing, electroencephalography, event-related potentials

## Abstract

The brain as a proactive system processes sensory information under the top-down influence of attention and prediction. However, the relation between attention and prediction remains undetermined given the conflation of these two mechanisms in the literature. To evaluate whether attention and prediction are dependent of each other, and if so, how these two top-down mechanisms may interact in sensory processing, we orthogonally manipulated attention and prediction in a target detection task. Participants were instructed to pay attention to one of two interleaved stimulus streams of predictable/unpredictable tone frequency. We found that attention and prediction interacted on the amplitude of the N1 ERP component. The N1 amplitude in the attended/predictable condition was larger than that in any of the other conditions. Dipole source localization analysis showed that the effect came from the activation in bilateral auditory areas. No significant effect was found in the P2 time window. Our results suggest that attention and prediction are dependent of each other. While attention might determine the overall cortical responsiveness to stimuli when prediction is involved, prediction might provide an anchor for the modulation of the synaptic input strengths which needs to be operated on the basis of attention.

## INTRODUCTION

Recent theories of sensory processing consider the brain as a proactive system which adapts quickly to the environment. Neurons in the sensory cortices can undergo short-term, task-dependent, and context-specific changes in receptive field properties when attention and prediction are involved (Fritz et al., 2003, 2007, 2008). Such adaptive plasticity driven by attention and prediction can be the underlying mechanism for the optimization ofperception.

Attention is suggested to have a global effect on perception at an early stage of sensory processing. Electroencephalography (EEG) studies revealed the neuronal consequences of attention on event-related potentials (ERPs), particularly the enhancement of the N1 (Hillyard et al., 1973; Alcaini et al., 1994; Lange et al., 2003, 2006; Lange and Röder, 2006). This may result from changes in the selectivity of neurons in the sensory cortex (Chawla et al., 1999; Kastner et al., 1999; Ahveninen et al., 2006). Specifically, research showing that the auditory N1 response is modulated by task demands and notched-noise masking suggests that the spectrotemporal receptive fields of neurons are tuned according to attentional manipulations (Kauramäki et al., 2007), as attention excites neurons responsive to attended features and inhibits neurons responsive to unattended features (Fritz et al., 2003, 2007, 2008; Jääskeläinen et al., 2007). Neurocomputational studies demonstrated that attention may function via optimizing the synaptic gain to represent the precision of sensory information during hierarchical inference (Feldman and Friston, 2010).

Prediction, or the statistical regularity in the environment, is also suggested to modulate the early stage of sensory processing, albeit its effect on ERPs manifests as a suppression of the N1 (Lange, 2013). Prediction-related N1 suppression was demonstrated when participants had foreknowledge of the upcoming stimuli (Schafer and Marcus, 1973; Schafer et al., 1981; Lange, 2009; SanMiguel et al., 2013; Timm et al., 2013). The predictive coding model postulates that the prediction effect indexes the difference in neurocomputational demand for predictable/unpredictable information (Friston, 2005; Egner et al., 2010). Specifically, it indexes how much of the sensory input cannot be accounted for by the internal model. Moreover, prediction was reported to alter the contrast gain of sensory evidence accumulation (Melloni et al., 2011; Rohenkohl et al., 2012). Neurophysiologically, this is reflected in sharper sensory representations where the reduction of neuronal activity is smaller in neurons responsive to predictable features than in neurons responsive to unpredictable features (Kok et al., 2012a).

Despite their ERP effects being opposite, the relation of attention and prediction remains undetermined. This might be due to the conflation of these two mechanisms in the literature, where attention and prediction were often treated as the same concept (Corbetta and Shulman, 2002). However, attention and prediction can rely on orthogonal sources of information (Summerfield and Egner, 2013). While attention operates on the basis of motivational relevance, prediction operates on the basis of prior likelihood (Summerfield and Egner, 2009). It is possible that attention and prediction are two independent mechanisms which may have antagonistic (Summerfield and Egner, 2009) or additive (Timm et al., 2013) effects on neuronal signals for sensory processing. Alternatively, attention and prediction may be dependent of each other. One possibility is that one of the two mechanisms is necessary for the other to take effect, but not the other way round (Kok et al., 2012b). Another possibility is that such dependency is bidirectional, with both attention and prediction being necessary to modulate sensory processing.

To examine the relation between these two top-down mechanisms, we orthogonally manipulated attention and prediction in a target detection task. Participants were instructed to pay attention to one of two interleaved stimulus streams of predictable/unpredictable tone frequency. Using EEG, we quantified N1 and P2 as dependent variables given that the former is involved in auditory perception and the latter is suggested to reflect the comparison between the sensory input and the internal model (Evans and Federmeier, 2007; Costa-Faidella et al., 2011). The design allowed us to evaluate whether attention and prediction are dependent of each other, and if so, how these two top-down mechanisms may interact on sensory processing.

## MATERIALS AND METHODS

### PARTICIPANTS

Sixteen healthy volunteers (average age 28; six males; all right-handed) with no history of neurological, psychiatric, or hearing impairments as indicated by self-report participated in the experiment. Participants gave written informed consent and were paid for participation. Ethical approval was granted by the Comité de Protection des Personnes (CPP) Ile de France II. The experiment conforms with The Code of Ethics of the World Medical Association (Declaration of Helsinki).

### STIMULI

Sinusoidal tones with a loudness of 80 phons (i.e., 80 dB for tones of 1000 Hz) were generated using Matlab. The duration of each tone was 50 ms (including 5 ms rise/fall times). The frequency of each tone was within the range of 261.626–493.883 Hz and 2093.000–3951.070 Hz, matching the absolute frequency of two sets of seven natural keys on a modern piano (low frequency set: C4 D4 E4 F4 G4 A4 B4; high frequency set: C7 D7 E7 F7 G7 A7 B7). Within each frequency set, the predictable/unpredictable stimulus streams were created. The predictable stimulus stream consisted of 400 pairs of tones. Each pair of tones was arranged in ascending order with the second tone being two natural keys higher than the first tone (e.g., C4-E4; G7-B7). The unpredictable stimulus stream consisted of 400 pairs of tones. Each pair of tones was arranged in random order with the second tone being any tone except the repetition of the first tone (e.g., C4-F4; G7-F7). The tones in each condition were of equal variability in frequency.

**FIGURE 1:**
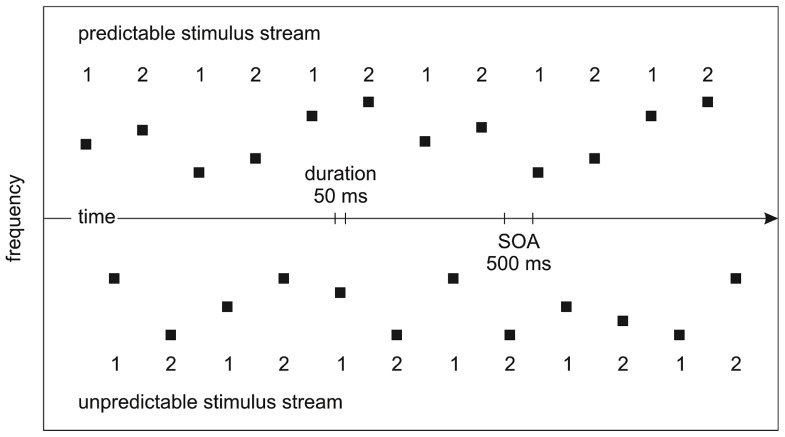
**Experimental design.** The predictable/unpredictable stimulus streams from different frequency sets were interleaved. The numbers indicate the tones being the first/second tones in each pairs of tones. Unaware of the manipulation of prediction on tone frequency, participants were instructed to pay attention to one of the stimulus streams in different blocks where tones of attenuated loudness may appear.

The predictable/unpredictable stimulus streams from different frequency sets were interleaved to allow for the efficient manipulation of attention on the two stimulus sets. To counterbalance the mapping between predictable/unpredictable stimulus streams and high/low frequency sets, half of the participants were presented with a low-frequency predictable stimulus stream interleaved with a high-frequency unpredictable stimulus stream and half of the participants were presented with a high-frequency predictable stimulus stream interleaved with a low-frequency unpredictable stimulus stream. To counterbalance the sequential relation of the predictable/unpredictable stimulus streams, half of the blocks started with the predictable stimulus stream and half of the blocks started with the unpredictable stimulus stream. A stimulus onset asynchrony (SOA) of 500 ms was used (**Figure 1**). 10% of the tones in one of the two stimulus streams served as targets attenuated in loudness by 20 dB, independent of whether they were the first/second tones in each pair of tones. Stimulation was created individually for each participant.

### PROCEDURES

Participants were presented with a total of eight blocks of 100 pairs of tones via headphones (Sennheiser PX200), with each block including 50 predictable pairs of tones and 50 unpredictable pairs of tones. Unaware of the manipulation of prediction, participants were instructed to pay attention to one of the stimulus streams (i.e., high/low frequency) in different blocks where tones of attenuated loudness may appear. To monitor whether participants followed the instructions correctly, participants were required to press a key when they detected a softer tone, which randomly occurred 10 times in each block. No practice session was provided. Block order was counterbalanced across participants. The experiment was administered conjointly with another EEG experiment which is to be reported elsewhere.

The stimuli of interest were the second tones in each pair of tones, which can be attended/predictable, attended/unpredictable, unattended/predictable, and unattended/unpredictable. Note that the manipulations of attention and prediction were both introduced on the basis of tone frequency. Moreover, all stimuli were presented binaurally. Therefore, there was no spatial effect in the current study.

### DATA RECORDING AND ANALYSIS

#### EEG recording and pre-processing

EEG was recorded with 64 active electrodes (actiCAP, Brain Products GmbH, Germany) conforming to the international 10–10 system. The sampling rate was 500 Hz. No online/offline filter was used. The data was recomputed to average reference. Target stimuli and the first stimuli following target stimuli were removed. Epochs extended from -100 to 500 ms relative to stimulus onset, using a 100 ms pre-stimulus baseline. Ocular artifact correction was conducted with independent component analysis in EEGlab (Delorme and Makeig, 2004). Epochs containing voltage deviations exceeding ±80 μV relative to baseline at any of the electrodes were rejected. The trial numbers after artifact rejection in each condition are listed in **Table 1**.

**Table 1:** Mean and range of trial numbers after artifact rejection in each condition.

	Attended predictable	Attended unpredictable	Unattended predictable	Unattended unpredictable
Mean	175.38	171.38	171.81	173.50
Range	165–182	141–187	142–188	161–181

#### ERP analysis

ERP analysis was based on a temporal principal component analysis (PCA) in SPSS 20. The temporal PCA statistically decomposes the ERP waveforms into constituent building blocks, which affords objective data-driven ERP component measures when compared to the conventional peak-picking methods (Kayser and Tenke, 2003; Dien et al., 2005; Kayser and Tenke, 2006; Dien, 2012). Moreover, it is not susceptible to the influences of high-frequency noises and low-frequency drifts in the data as the conventional peak-picking methods (Luck, 2005). Covariance matrix and Promax rotation were used. All components accounting for a total of 99% of the variance (maximum iterations for convergence = 500) were included in the rotation (Promax Kappa = 4). The temporal decomposition provided a set of component loadings reflecting the contribution of each time point on the temporal principal components. The component loadings were used to derive component scores which represent the magnitude of neural activity within the time windows of interest. We identified N1 and P2 by selecting components showing typical N1 and P2 latency and topography. Electrodes showing the largest N1 component score (i.e., F2) and P2 component score (i.e., Cz) were considered as representative electrodes for the components of interest. Therefore, their component scores were used as input for the 2 (attended/unattended) × 2 (predictable/unpredictable) repeated measures analysis of variance (ANOVA).

## RESULTS

### BEHAVIOURAL DATA

Overall, participants’ performance in the target detection task was at ceiling (Hit: mean = 0.96, SD = 0.03; False alarm: mean < 0.01, SD < 0.01; RT: mean = 539.34, SD = 62.21), confirming that participants followed the instructions correctly. The ceiling performance rendered it unlikely that participants’ attention alternated between the two stimulus streams within blocks, which would have brought down participants’ performance. There was no difference between participants’ performance when they attended to predictable/unpredictable stimulus stream [Hit: *t*(15) = 0.30, *p* = 0.77; False alarm: *t*(15) < 0.01, *p* = 1.00; RT: *t*(15) = 0.32, *p* = 0.75]. The equivalent performance across blocks excluded the possibility that task difficulty may be a confounding factor in the current study.

### ERP DATA

**FIGURE 2:**
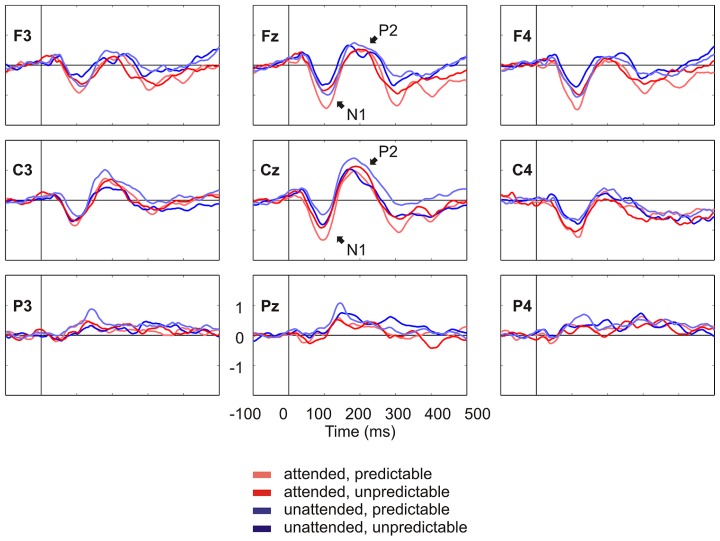
**Grand average ERPs on nine representative electrodes (F3, Fz, F4, C3, Cz, C4, P3, Pz, P4) lowpass filtered at 30 Hz for visual presentation purposes.** N1 and P2 in the grand average ERPs are denoted.

**FIGURE 3:**
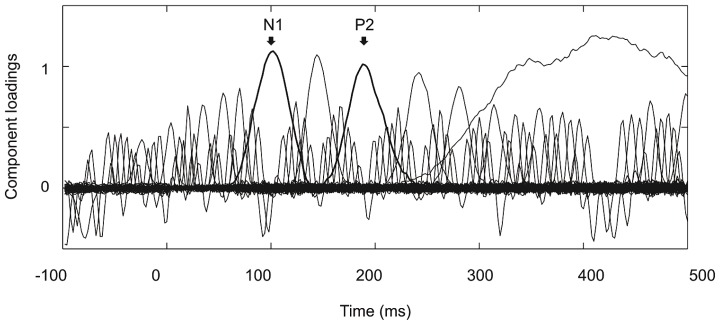
**Component loadings of 111 components in the temporal PCA.** Components clearly corresponding to N1 and P2 in the grand average ERPs are marked with thick lines.

**FIGURE 4:**
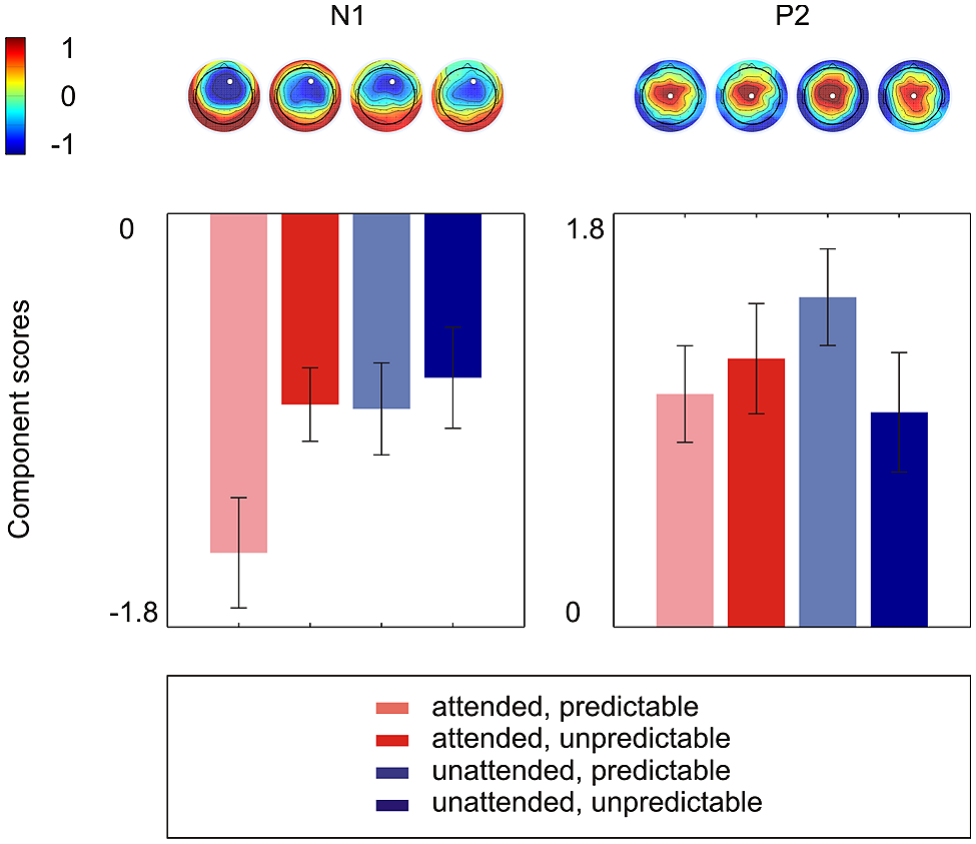
**The N1 and P2 component scores on electrodes showing the largest responses (N1: F2; P2: Cz) and the topographical distribution of components in each condition.** Error bars depict one standard error above and below the mean.

**Figure 2** shows the grand average ERPs on nine representative electrodes (F3, Fz, F4, C3, Cz, C4, P3, Pz, P4). N1 with a frontocentral distribution and P2 with a central distribution are evident. **Figure 3** shows the component loadings of 111 components in the temporal PCA. The temporal PCA yielded components clearly corresponding to N1 and P2 in the grand average ERPs. **Figure 4** shows the N1 and P2 component scores on electrodes showing the largest responses (N1: F2; P2: Cz) and the topographical distribution of the components in each condition.

#### N1

**FIGURE 5:**
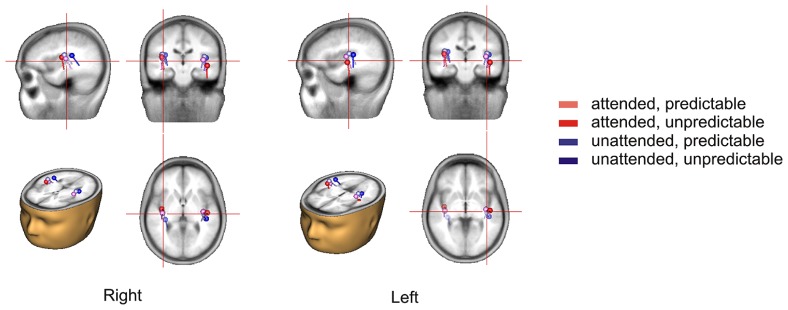
**Source locations of the N1 response in each condition based on the topographical distribution of the component in the temporal PCA**.

The 2 × 2 repeated measures ANOVA showed a significant main effect of attention with attended stimuli triggering enhanced activity compared to unattended stimuli [*F*(1,15) = 6.50, *p* < 0.05] and a marginally significant main effect of prediction with predictable stimuli triggering enhanced activity compared to unpredictable stimuli [*F*(1,15) = 3.40, *p* < 0.10]. More importantly, there was a significant attention × prediction interaction [*F*(1,15) = 4.61, *p* < 0.05]. *Post hoc* comparisons showed that the component score was larger for attended stimuli than for unattended stimuli in the predictable condition [*t*(15) = -3.42, *p* < 0.01] but not in the unpredictable condition [*t*(15) = -0.60, *p* = 0.56] and that the component score was larger for predictable stimuli than for unpredictable stimuli in the attended condition [*t*(15) = -2.40, *p* < 0.05] but not in the unattended condition [*t*(15) = -0.63, *p* = 0.54]. Moreover, the component score for attended/predictable stimuli was larger than that for unattended/unpredictable stimuli [*t*(15) = -2.44, *p* < 0.05]. In other words, the component score in the attended/predictable condition was larger than that in any of the other conditions. To examine the neural origin of the effects of attention and prediction, the component scores on all electrodes for N1 were further imported into BESA Research 6.0 for dipole source localization (Scherg and von Cramon, 1986). A four-shell ellipsoidal head model was used. Two equivalent current dipoles were randomly seeded and freely fitted. The analysis showed that all four conditions had sources close to each other in the bilateral auditory areas (**Figure 5**). The average Talairach coordinates across the four conditions (left, *x*: -40.5, *y*: -20.9, *z*: 6.5; right, *x*: 37.0, *y*: -18.9, *z*: 13.5) are close to the approximate location of the Heschl’s gyrus (e.g., the average limits in Penhune et al., 1996 are for the left *x*: -32.5 to -62.3, *y*: -33.2 to -4.9, *z*: 1.4 to 14.2 and for the right *x*: 34.7 to 58.2, *y*: -32.8 to -4.7, *z*: 1.1 to 17.7). The two dipole model had residual variances of 1.22% (attended/predictable), 1.62% (attended/unpredictable), 2.51% (unattended/predictable), and 2.45% (unattended/unpredictable). The result suggests that the effects of attention and prediction are based on the activation of neuronal populations in the bilateral auditory areas.

### P2

In the 2 × 2 repeated measures ANOVA, neither the main effect of attention [*F*(1,15) = 0.44, *p* = 0.52] nor the main effect of prediction [*F*(1,15) = 0.70, *p* = 0.42] was significant. The attention × prediction interaction was marginally significant [*F*(1,15) = 4.15, *p* = 0.06]. However, there was no significant effect in Tukey LSD *post hoc* comparisons (Attention effect in predictable condition: *t*(15) = -1.99, *p* = 0.07; Attention effect in unpredictable condition: *t*(15) = 1.08, *p* = 0.30; Prediction effect in attended condition: *t*(15) = -0.61, *p* = 0.55; Prediction effect in unattended condition: *t*(15) = 1.84, *p* = 0.09).

## DISCUSSION

The orthogonal design in the current study allowed us to evaluate whether attention and prediction are dependent of each other, and if so, how these two top-down mechanisms may interact for the optimization of perception. We found that attention and prediction interacted on the amplitude of the N1 ERP component generated in the auditory cortices. The N1 amplitude in the attended/predictable condition was larger than that in any of the other conditions. The relatively early latency of the interaction between these two variables is in line with the idea that attention and prediction can synergistically enhance perceptual analysis through top-down pathways (Doherty et al., 2005; Chaumon et al., 2008). Furthermore, it seems that attention and prediction rely on each other to form a tonically maintained set which can selectively include/exclude sensory input for further processing (Hillyard et al., 1973; Kok et al., 2012b). In other words, both attention and prediction are involved in the optimization of sensory processing.

Precious research reported that neurons in the sensory cortices can undergo short-term, task-dependent, and context-specific changes in the receptive field properties (Fritz et al., 2003, 2007, 2008). Such changes in the selectivity of sensory cortex neurons are believed to be the mechanism underlying the optimization of perception (Ahveninen et al., 2006; Kauramäki et al., 2007; Kok et al., 2012a). Extending the idea, our results further suggest that both attention and prediction are needed for such rapid plasticity to occur. Specifically, one may speculate that while attention determines the overall cortical responsiveness to stimuli (Lakatos et al., 2008; Busch et al., 2009; Schroeder and Lakatos, 2009), prediction serves as an anchor for the selective modulation of synaptic input strengths. An interesting question for future research is whether it is possible to disentangle the synergistic neuronal effects of attention and prediction on sensory processing.

The prediction-dependent attention effect demonstrates, for the first time, that attention alone may not be able to reshape perceptual inferences. It is unknown whether the prediction participants formed in the current study was implicit or explicit. However, it seems that prediction, be it implicit or explicit, is needed for attention effects to occur. At first glance, this idea seems difficult to reconcile with previous studies showing that attention can selectively modulate the response of neuronal subpopulations that prefer the attended stimulus features (Martinez-Trujillo and Treue, 2004; Liu et al., 2007). However, it is noteworthy that, in previous studies, attended stimulus features were also predictable stimulus features, making it difficult to disentangle the influence of prediction on attention effects. Our finding of the prediction-dependent attention effect suggests that prediction may be an important construct for attention.

The attention-dependent prediction effect, on the other hand, sheds light on the dynamic influence of attention on prediction. Notably, using functional magnetic resonance imaging (fMRI) and visual stimuli, Kok et al. (2012b) reported an approach of manipulating attention and prediction that is similar to ours. In the attended condition, our finding of prediction-related enhancement of the N1 amplitude from the auditory cortices was consistent with Kok et al.’s finding of prediction-related enhancement of activation in early visual cortex. Such enhancement of neuronal responses confirm the notion that the engagement of attention may increase the weighting of sensory information, resulting in enhanced neuronal responses to predictable stimuli relative to unpredictable stimuli (Rao, 2005). The enhanced N1 is nicely in line with the predictive coding theory of sensory processing. The predictive coding theory suggests that perception entails the propagation of signals from two distinct types of neuronal populations, the representation neurons, and the prediction error neurons (Friston, 2005, 2009; Egner et al., 2010). Although there was some debate in recent neurocomputational research concerning which type of neurons is modulated by attention (Spratling, 2008, 2010; Feldman and Friston, 2010), it was shown that both models presume enhanced responses to predictable stimuli relative to unpredictable stimuli in the attended condition. In contrast, in the unattended condition, our null result was inconsistent with Kok et al.’s finding of prediction-related suppression of activation in early visual cortex. While caution should be taken for the interpretation of our null result, we speculate that the discrepancy may be related to the different manipulations of attention and prediction.

Concerning the manipulation of attention, the discrepancy may be explained by a competition hypothesis borrowed from research on mismatch negativity (MMN). Mismatch negativity is generated to violations of abstract stimulus prediction rules (Tervaniemi et al., 1994; Paavilainen et al., 1999), reflecting the detection of deviant stimuli independent of attention (Näätänen, 1990; Näätänen et al., 2007). Importantly, a competition hypothesis suggests that, although attention does not affect the deviant detection process *per se*, it may influence the sensory information reaching the deviant detection process (Sussman et al., 2003). For example, when certain stimulus features are attended in a competing stimulus stream, the detection of stimuli deviating in certain stimulus features can be accordingly abolished in the unattended channel, leading to the absence of the MMN.

We suggest that whether the prediction effect appears in the unattended condition depends on the degree of such competition for cognitive resources. In the current study, attention was manipulated in the “filtering” manner. In this case, participants may inhibit the processing of unattended stimuli stream at an early stage to get rid of unnecessary information. In the study of Kok et al. (2012b), on the contrary, attention was manipulated in the “probabilistic” manner. In this case, participants may not inhibit the processing of unattended stimuli until the level concerning behavioral output. Consequently, the competition for cognitive recourses may be too weak to eliminate the possibility that unpredictable stimuli automatically attract attention, resulting in enhanced neuronal responses (Larsson and Smith, 2012). The same line of argumentation holds for previous studies demonstrating prediction-related suppression effects, in which participants’ attention was either not controlled (Schafer and Marcus, 1973; Stefanics et al., 2010; Besle et al., 2011) or directed away from the manipulation of prediction using a cover task (Schafer et al., 1981; Summerfield et al., 2008; Lange, 2009; Todorovic et al., 2011; Kok et al., 2012a; Todorovic and de Lange, 2012). Given that participants’ attention still stayed focused on the stimuli in these tasks, it is difficult to exclude the possibility that the observed prediction-related suppression effects reflected the enhanced neuronal responses to unpredictable stimuli.

Alternatively, whether the prediction effect appears in the unattended condition may depend on the particular manipulation of prediction. In the current study, the unpredictable condition consisted of stimuli randomly selected from a given frequency set. In other words, it is difficult for participants to form a specific prediction about the frequency of the upcoming stimuli in the first place. Therefore, the activation in sensory cortex may reflect exclusively the neuronal signals triggered by the presence of unpredictable stimuli. In the study of Kok et al. (2012b), on the contrary, the unpredictable condition consisted of stimuli which were presented as oddballs that violated participants’ prediction. Consequently, the activation in sensory cortex may reflect not only the neuronal signals triggered by the presence of unpredictable stimuli but also the neuronal signals triggered by the absence of predictable stimuli. Such increase in the neuronal signals in the unpredictable condition may be the reason why prediction-related suppression can be observed.

On the other hand, no significant effect was found in the P2 time window. At best, there was a marginally significant interaction between attention and prediction. While caution should be taken in interpreting the results, the pattern of the interaction suggests that attention reversed the direction of prediction effects on the P2. In the attended condition, predictable stimuli suppressed the P2. In the unattended condition, predictable stimuli enhanced the P2. Interestingly, the pattern is opposite to the findings of Kok et al. (2012b), where prediction-related hemodynamic responses in early visual cortex were enhanced when the stimuli were attended and suppressed when the stimuli were unattended. More research is needed to examine whether the interaction between attention and prediction may change over time and how such dynamics manifest in hemodynamic responses.

Overall, our findings suggest that future research on attention and its relation to prediction needs to differentiate between different manipulations of attention and prediction. Moreover, our results confirm the importance of incorporating the modulatory effect of attention in the predictive coding theory. The predictive coding theory postulates that the prediction effect indexes the difference in neurocomputational demand for predictable/unpredictable information (Friston, 2005; Egner et al., 2010). Specifically, it is suggested that predictable information, relative to unpredictable information, elicits smaller prediction error signals. The smaller prediction error signals are associated with reduced connectivity within a hierarchical cortical network (Garrido et al., 2007, 2009), which accounts for the observations of prediction-related suppression in evoked responses (Wacongne et al., 2011). The predictive coding theory seems contradictory to our findings of significant prediction-related N1 enhancement in the attended condition and the lack of prediction-related N1 suppression in the unattended condition, unless the modulatory effect of attention is taken into consideration. Recent computational models demonstrated that attention emerges naturally in a similar Bayes-optimal scheme as the inference about the precision of the causes of sensory input (Friston, 2009; Feldman and Friston, 2010). It is, thus, possible that the prediction effect does not reflect merely the different prediction error signals. Rather, it reflects how the precision of the causes of sensory input is weighed against the prediction error signals. In other words, it is the attention effect, rather than the prediction effect, that is dominant here. In the attended condition, the precision of the causes of sensory input increases. The increased precision may manifest as an imaginary tuning curve with narrower bandwidth. Moreover, it may outweigh the prediction error signals to predictable input over the prediction error signals to unpredictable input. This is when the prediction-related enhancement can be observed. In the unattended condition, on the other hand, the precision of the causes of sensory input decreases. It can manifest as an imaginary tuning curve with wider bandwidth. In this case, the amount of the decrease in precision may determine to what extent the prediction error signals to unpredictable input can be canceled out. The larger the decrease in precision, the more it cancels out the prediction error signals to unpredictable input. At some point, the prediction error signals to unpredictable input would become really small, rendering the prediction-related suppression negligible. A real life analogy is that, when one pays minimum amount of attention to the stimuli, the boundary between the unpredictable input and the predictable input is of little importance. This mechanism could be tested in detail in future research.

## Conflict of Interest Statement

The authors declare that the research was conducted in the absence of any commercial or financial relationships that could be construed as a potential conflict of interest.
