# Corrigendum: Both attention and prediction are necessary for adaptive neuronal tuning in sensory processing

**DOI:** 10.3389/fnhum.2017.00255

**Published:** 2017-05-19

**Authors:** Yi-Fang Hsu, Jarmo A. Hämäläinen, Florian Waszak

**Affiliations:** ^1^Université Paris Descartes, Sorbonne Paris CitéParis, France; ^2^CNRS, Laboratoire Psychologie de la Perception, UMR 8242Paris, France; ^3^Department of Psychology, University of JyväskyläJyväskylä, Finland

**Keywords:** attention, prediction, sensory processing, electroencephalography, event-related potentials

1. In the original article, there was **a typo where participants' average age is incorrect. It should be “29” not “28.”**
  A correction has been made to Materials and Methods, Participants, Paragraph 1:
  Sixteen healthy volunteers (**average age 29**; six males; all right-handed) with no history of neurological, psychiatric, or hearing impairments as indicated by self-report participated in the experiment. […].
2. In the original article, there was **a typo where “previous” was misspelled as “precious.”**
  A correction has been made to Discussion, Paragraph 2:
  **Previous** research reported that neurons in the sensory cortices can undergo short-term, task-dependent, and context-specific changes in the receptive field properties (Fritz et al., 2003, 2007, 2008). […].

The authors apologize for this error and state that this does not change the scientific conclusions of the article in any way.

## Conflict of interest statement

The authors declare that the research was conducted in the absence of any commercial or financial relationships that could be construed as a potential conflict of interest.

